# “It Gave Me Hope” Experiences of Diverse Safety Net Patients in a Group Acupuncture Intervention for Painful Diabetic Neuropathy

**DOI:** 10.1089/heq.2020.0004

**Published:** 2020-05-22

**Authors:** Rhianon Liu, Trilce Santana, Dean Schillinger, Frederick M. Hecht, Maria T. Chao

**Affiliations:** ^1^ UCSF Osher Center for Integrative Medicine, UCSF, San Francisco, California, USA.; ^2^ Division of General Internal Medicine, UCSF, San Francisco, California, USA.

**Keywords:** diabetes, acupuncture, health disparities, quality of life, pain, painful diabetic neuropathy

## Abstract

**Purpose:** To explore the experiences of living with painful diabetic neuropathy (PDN) and with a group acupuncture intervention in a sample of low-income, diverse patients.

**Methods:** We conducted a randomized clinical trial of a 12-week group acupuncture intervention for PDN. Data included validated measures of patient-reported outcomes, including pain and quality of life (QOL), as well as semistructured qualitative interviews about participants' experiences with PDN and the intervention. Interview transcripts were coded and analyzed using an inductive thematic framework.

**Results:** We recruited 40 participants from diverse racial/ethnic backgrounds from a public hospital and conducted in-depth qualitative interviews with a subset of 17 participants. Participants randomized to acupuncture experienced greater decreases in pain compared with usual care as well as improved QOL. In interviews, they described a myriad of socioeconomic and personal life stressors that compounded the significant suffering and disability brought on by PDN. Those who received acupuncture were able to decrease reliance on pain medication, improve their sleep and daily function, reduce stress, and engage more with their own self-care. They noted that the acupuncture intervention also gave them hope in the face of their chronic disease.

**Conclusion:** Acupuncture is a valuable adjunct treatment for low-income and marginalized populations with PDN. In addition to reducing pain and improving QOL, acupuncture may offer powerful benefits by increasing patient activation and hope.

## Introduction

Neuropathy is one of the most disabling and costly complications of diabetes,^1^ and greatly impairs patients' quality of life (QOL).^2,3^ Significant disparities exist in the prevalence, management, and outcomes of diabetes in general and of diabetic neuropathy in particular. Racial/ethnic minorities have higher rates of diabetes, less access to care (including screening and counseling), and higher diabetes-related morbidity and mortality compared with non-Hispanic whites.^4^ Despite a higher rate of diabetic complications,^5^ racial/ethnic minorities and low-income patients are less likely to be correctly diagnosed with neuropathy,^6^ and suffer more sequelae such as loss of protective sensation, foot ulcers, and lower limb amputations.^7,8^

Current treatments for painful diabetic neuropathy (PDN) are limited, and most are aimed at analgesia. Serotonin/norepinephrine reuptake inhibitors, anticonvulsants, and topical capsaicin are considered superior to placebo for short-term pain control, although there is a dearth of high-quality evidence.^9^ Up to 40% of patients are treated with opiates,^10^ despite limited efficacy and significant side effects, including somnolence, dizziness, and nausea.^11^

Prior research has found that patients with diabetes commonly use complementary health approaches.^12,13^ One emerging nonpharmacologic intervention for neuropathic pain is acupuncture. Acupuncture is now widely recognized as an effective treatment for chronic pain,^14^ and a recent review concluded that it is likely beneficial for peripheral neuropathy,^15^ although more evidence is needed. Existing research demonstrates that when available, diverse and low-income patients avail themselves of integrative therapies,^16,17^ including acupuncture.^18,19^ Group acupuncture (a method of delivery in which multiple people receive acupuncture in a common area) can reduce cost and facilitate access for medically marginalized populations.^20,21^ A recent study found that 30% of participants randomized to group acupuncture had clinically relevant reductions in back, neck, or osteoarthritis pain, although noninferiority to individual acupuncture was not established.^22^

To better understand how PDN impairs QOL, patient perspectives are needed. A recent study demonstrates that PDN impacts patients' physical function, daily life, psychological well-being, and sleep.^23^ However, there is still a need to give voice to vulnerable patient groups who bear a disproportionate burden of disease and are typically underrepresented in research.^24^ The aim of this analysis is to explore participants' experiences living with PDN and with group acupuncture, to guide the development of more effective treatments for those most affected.

## Methods

### Study design

Our research team conducted a randomized clinical trial that demonstrated feasibility and acceptability of a group acupuncture intervention for PDN among a sample of linguistically and racially diverse safety net patients.^25^ Using a mixed-methods, embedded design, qualitative data were collected to heighten our understanding of quantitative outcomes. Detailed methodology and quantitative patient-reported outcomes have previously been described.^25^ Here, we report qualitative data from a subsample of study participants to further describe their perspectives in the context of quantitative, patient-reported outcomes (e.g., pain and QOL). The Institutional Review Board at the University of California San Francisco approved all study procedures.

### Participants

As part of the larger study, participants were recruited from outpatient clinics at the Zuckerberg San Francisco General Hospital and Trauma Center, an urban public hospital serving diverse patients (70% racial/ethnic minorities, 80% publicly insured or uninsured). Eligible participants were aged ≥18 years, English or Spanish speaking, and diagnosed with type 2 diabetes with neuropathic pain intensity of ≥4 on an 11-point (0–10) numerical rating scale. Participants from the larger study were invited to take part in a longer qualitative interview; data reported here are from 17 participants who had availability and interest.

### Data collection and measures

Study participants provided written consent and completed interviewer-administered assessments at four time points: baseline and weeks 6, 12, and 18. At each time point, we collected patient-reported outcomes such as pain and QOL using validated instruments.^26,27^ At baseline, we also collected descriptive sociodemographic and clinical characteristics.

Assessments at weeks 12 and 18 included semistructured interview questions about patients' experiences with study procedures; physical, emotional, or lifestyle changes since beginning of the study; and life stressors and supports (interview guide available upon request). Participants who were randomized to the study intervention were asked additional questions about their experiences with acupuncture treatment. Qualitative interviews were digitally recorded.

### Analysis

Interviews were transcribed verbatim, and Spanish-language interviews were translated into English. Codebook thematic analysis was done using an inductive approach.^28^ Two authors (R.L., T.S.) independently coded an initial four interviews using an open coding framework, and then jointly developed a codebook. The two authors then applied this coding scheme to four additional interviews, and conferred with the primary investigator (M.T.C.) to reach consensus about the final codebook. The remaining nine interviews were then coded by one author (R.L.). Coding was done using Dedoose software,^29^ a web-based application designed for management and analysis of mixed-methods research data. Once all interviews were coded, codes were arranged into broader themes pertaining to the study questions.

Descriptive statistics, including frequencies, percentages, means, and standard deviations, were calculated from survey data using Stata statistical software.^30^

## Results

### Study sample

Participants of the qualitative study (*n*=17) were an average age of 59.4 years; 18% African American/black, 12% Asian/Pacific Islander, 47% Latino, and 24% non-Latino white; 65% were born outside the United States; all had an annual income of <$35,000. Participants who completed qualitative interviews were not significantly different than the full study sample (Table 1).

**Table 1.** Characteristics of Study Participants

Characteristic	Qualitative subsample (*n*=17) *N* (%)	Full sample (*n*=40) *N* (%)
Age, years (mean±SD)	59.4±9.9	58.8±11.1
Female	8 (47)	20 (50)
Race/ethnicity		
African American/black	3 (18)	8 (20)
Asian/Pacific Islander	2 (12)	4 (10)
Latino	8 (47)	20 (50)
Non-Latino white	4 (24)	7 (18)
Born outside the United States	11 (65)	23 (58)
High school education or less	8 (47)	17 (43)
Primary language, Spanish	6 (35)	20 (50)
Insurance		
Uninsured	—	3 (8)
Public insurance	15 (88)	33 (83)
Employment		
Unemployed	6 (35)	13 (33)
Retired	5 (29)	13 (33)
On disability	3 (18)	4 (10)
Annual household income <$35K	17 (100)	37 (93)

SD, standard deviation.

### Living with PDN

Participants described a wide range of painful sensations resulting from diabetic neuropathy, using words such as “ache,” “throbbing,” and “needle-sharp,” as well as “numbness” and loss of sensation. At baseline, participants reported an average pain intensity of 5.3 on an 11-point numerical rating scale.^25^ Many stated that onset of pain or numbness was unpredictable, interfering with their activities of daily living. One participant, with a baseline average pain of 5, described how neuropathy makes him reluctant to walk:

“It seems like the first thing that happens is I get dizzy, or I get off balance. The sidewalks are very uneven in the city and, you know, you don't lift your feet high enough. So, I think that's a direct thing of neuropathy and it stops me from walking as much, although I still try, but I ha[ve] to be very, very conscious. And you shouldn't be very, very conscious of walking, you should walk. If I'm not, I'm gonna fall down.”—Male, age 62, acupuncture group, 1-point decrease in pain

Another common concern was that pain or other unpleasant sensations resulting from neuropathy significantly impaired participants' sleep. On a scale of 0–100, participants reported an average sleep disturbance of 49.4. One participant, with a baseline sleep disturbance of 51.2, mentioned,

“It's pretty uncomfortable, like you try to sleep and then you fall asleep for 20 minutes and then you wake up because of the pain coming and you try to move your legs. So, even my children don't want to lie down with me for more than 10–15 minutes because I move too much.”—Female, age 45, usual care group, no change in pain

For these individuals, neuropathy has made essential functions such as walking or sleeping challenging.

### Quality of life

PDN was only one of many stressors affecting participants in this study. In addition to their health challenges, they described lack of transportation, difficulty accessing health care teams, and limited social support. Many were caring for other ill or elderly family members themselves. Unstable housing was a common concern. One participant worried,

“Money is a weird one. I mean I have more money now than I probably ever really had, but as I live in one of the most expensive cities in the U.S., you get more money and all the prices go up… I would never be able to buy a house here. Even renting, I'm lucky to live where I live now, but if that ends, I don't know if I can stay in the city. Sometimes, those are big pressures.”—Male, age 62, acupuncture group, 1-point decrease in pain

At the root of many of these problems is poverty, as illustrated by one participant:

“After we pay bills, we have $200 left for two adults and a dog. It's not very much for the whole month. The last week of the month, we live on rice and beans and air.”—Female, age 67, acupuncture group, no change in pain

The competing demands and the struggle for life's basic necessities presented significant barriers to participants' ability to care for their diabetes.

### Impact of acupuncture on PDN

Overall, participants found acupuncture helpful for PDN. Most noted that although their symptoms were still present, they were significantly improved. Average pain intensity for participants randomized to group acupuncture decreased by 1.8 points over 12 weeks.^25^ One participant stated,

“My legs hurt a lot…but now it's not continuous but rather two or three days that it hurts, not like it was in the beginning when I came here. Nor is the cramping so strong. The soles of my feet and my hands would cramp very much…. anywhere I was – at home the same as on the street. But now they cramp but not to the degree that it was.”—Female, age 60, acupuncture group, 1-point decrease in pain

Some participants described decreased use of medications, such as opioids and benzodiazepines, while receiving acupuncture. One participant recalled,

“I have been prescribed hydrocodone for a couple of years now. And my doctor does not want to hand out pills, believe me, but she understands that I do need those. But I sort of hate taking all those things. With the side effects of those things, like with your bowel movements, it makes it really hard to go the bathroom, which is miserable. I have really, really cut back on those hydrocodone…I ha[ve] been able to cut back on that without being in intolerable pain.”—Male, age 62, acupuncture group, 1-point decrease in pain

Multiple participants also reported great improvement in their sleep. For one participant, who reported a 5-point improvement in sleep disturbance, the ability to sleep was transformative:

“I would get very distraught before because sometimes I only slept for 2 hours at night. When I started the acupuncture, I started to sleep and even now, if my attendant doesn't wake me up, I sleep 12 hours. He wakes me up to take medicine and to eat but I sleep a lot more, like I'm recovering from all of the years I didn't sleep.”—Female, age 67, acupuncture group, no change in pain

Participants (even those who reported less than average decrease in pain) consistently highlighted how acupuncture positively impacted the physical symptoms that were the most bothersome aspects of living with PDN. All participants randomized to acupuncture who were interviewed reported at least two areas of positive impact on their physical symptoms, such as decreased pain, fewer cramps, improved mobility, better balance, and better circulation. Benefits of acupuncture were not the same across all participants; some reported no changes in specific areas. For instance, one participant experienced decreased pain on the bottom of his feet, but no impact on the pain and numbness in his legs and hands, which he attributed to his arthritis (male, age 54, 1-point decrease in pain). Another participant reported feeling physically stronger and less numbness, but reported that acupuncture had not affected other symptoms (male, age 61, 5-point decrease in pain).

### Impact of acupuncture on QOL

Participants receiving acupuncture reported statistically significant improvement in QOL scores. Consistent with this, in interviews they described general positive life changes beyond control of specific PDN symptoms. For example, one participant reported an 18-point improvement in QOL physical function, from 11 to 29 on a 60-point scale. He stated:

“I'm not gonna say it's gonna cure you 100%, but it can bring back all your blood circulation and your movement and to help you get more energy from it.”—Male, age 43, acupuncture group, no change in pain

Other participants reported being more physically active, even in small ways. Another participant, who noted a 15-point QOL improvement in physical function, was able to improve his activity by choosing public transportation:

“I'm actually relying more on [public transit] to get about instead of driving—it is not that strenuous of an exercise getting on and off a bus and moving about the city, but it is better than—It's more exercise than, you know, previously. This is something new.”—Male, age 60, acupuncture group, 2-point decrease in pain

Psychological benefits were also reported. Participants generally found the treatments calming, and helpful for managing stress. Others noted an improvement in their overall mood.

“[Acupuncture] has done me a lot of good because it really has alleviated a lot of the pain and many things that I had. It has lifted my spirits because when I came here, I was doing really badly… Very very depressed. Very depressed.”—Female, age 60, acupuncture group, 1-point decrease in pain

Another participant described experiencing severe depression at the onset of the study, to the point where she was completely socially isolated. Over the course of the study, she resumed communication with her social worker and began communicating with her neighbors, even volunteering to babysit. The interviews revealed profound changes in patients' QOL that were not always captured by quantitative measures of symptoms.

### Participant engagement

In addition to attributing multiple physical and psychological benefits to acupuncture, participants reported improved engagement with caring for their disease. As one participant explained, simply understanding the diagnosis of neuropathy was a breakthrough:

“I only heard the word neuropathy, which I still am not sure how to pronounce correctly, maybe a month before I came here. I think I heard it on television, but just didn't relate it to anything that had to do with me. But when I started looking into it, I found that about half of the people with diabetes actually develop neuropathy. And so like that…I had discovered that what I had going on with my feet here, you know, was an official disease and other people knew about it.”—Male, age 60, acupuncture group, 2-point decrease in pain

Addressing the barrier of health literacy reduced feelings of isolation and helplessness. His QOL improved by 12 points, and he found renewed energy for other aspects of diabetes self-care:

“I have returned to be[ing] more conscious of the pain in my feet. Back when I was first diagnosed I attended a diabetic educational something with a lady that was the expert… At the end of the study, I returned to the things I already knew like put Vaseline into my—you know, making sure my feet is oiled and stuff like that.”—Male, age 60, acupuncture group, 2-point decrease in pain

Decreased physical pain in combination with supportive study staff impacted participants' motivation to be physically active and take more control of their health. Eleven participants (65%) reported that involvement with the study led them to make better health decisions such as exercising and improving their eating habits.

“Actually, I lost a significant weight from the last time. I lost about 6 pounds… And I exercise more. I walk up to about an hour and a half now… I'm on a [weight loss program]. So, kinda like eat what I have to eat, but only eat less.”—Male, age 43, acupuncture group, no change in pain.

Another participant who reported a 22-point improvement in QOL physical function also described feeling more hope, which she attributed to the therapeutic relationship with study staff:

“I would anticipate the joy of coming here because the [acupuncturists] were so kind…I felt more comfortable and trusting and it gave me hope. I was destroying myself at home little by little.”—Female, age 67, acupuncture group, no change in pain

This statement highlights the critical importance of the provider/patient relationship in treating the whole person, beyond simply reducing pain. In addition to increased hope and motivation, participants displayed greater awareness of their disease and its effect on their well-being. Taken together, these descriptions suggest that the experience of receiving acupuncture treatments as part of the study gave participants greater agency in their own disease trajectory.

## Discussion

Overall, participants found acupuncture beneficial for a range of symptoms common with PDN, including burning or aching pain, sensations of heat or cold, or numbness. This diversity of presentations partially explains why conventional treatments such as pain medications have limited efficacy in treating this condition. Acupuncture may hold unique promise as a treatment for PDN because of its potential to address patients' multifaceted pain experience, including physical symptoms as well as psychological well-being. Our findings are consistent with literature indicating that acupuncture is associated with reduced pain intensity and improved QOL for osteoarthritis,^31^ primary dysmenorrhea,^32^ and cancer-related pain.^33^

Assessment of study participants' overall QOL demonstrates that PDN or even diabetes in general represents only one of their major life stressors. Poverty and the resulting lack of transportation, stable housing, or healthy food options cause significant distress and prevent individuals from optimizing management of chronic health conditions such as diabetes. Participants described that in addition to pain management, acupuncture helped them increase physical function, manage stress, and improve their mood. Although acupuncture is primarily accessed by patients with more financial resources,^34^ our data suggest that it may offer powerful benefits for patients who face the additional stresses of poverty and structural inequality, defined as unequal status systematically rooted and perpetuated in social institutions (e.g., housing, health care, employment, education).^35^

Notably, participants became more engaged in caring for their diabetes through study participation. Patient engagement, patient activation, and self-efficacy are related concepts^36^ that as a group have been shown to improve health care outcomes and patient experiences.^37^ One definition of patient activation is patients' willingness and ability to take independent actions to manage their health and care.^37^ Regarding diabetes in particular, programs focused on patient empowerment or patient activation have been shown to reduce hemoglobin A1c, clinic visits, and all-cause mortality.^38–41^ Self-efficacy and patient activation are associated with diabetes self-management behaviors such as improved diet and exercise.^41,42^

Multiple participants also specifically mentioned hope, which is considered an essential component of resilient mental health in the field of psychology.^43^ Hope is also increasingly recognized as an important concept in medicine, including in the context of initiating new treatments.^43^ Hope may play an especially important role in patients who are suffering from chronic pain or other chronic conditions,^43,44^ including diabetes.^45^

Prior reviews indicate that acupuncture is a promising intervention for diabetes and related symptoms, such as improvements in glycemic control and neuropathic pain.^15,46^ An important contribution of our study is the focus on diverse safety net patients—including individuals born outside the United States—who suffer most from diabetes and its complications. Racial/ethnic minorities have lower levels of patient activation, compared with non-Hispanic whites, associated with greater unmet medical needs.^47^ A meta-analysis of complementary and alternative medicine trials found that increased hope and patient activation were commonly reported outcomes.^48^ More research would be beneficial to explore how acupuncture or other integrative health modalities might impact patient empowerment and hope in marginalized, non-U.S.-born, and low-income populations.

### Limitations

The following limitations should be noted in interpreting our study findings. Our study participants comprised a small sample of primarily low-income patients, many of whom were non-U.S.-born recruited from public health, safety net clinics, which limits the generalizability of our study. Participants enrolled in a study of group acupuncture for PDN and likely had a propensity toward complementary health approaches. Prior research indicates that positive expectations are associated with better treatment outcomes.^49^ In addition, participants who had a beneficial experience with the study may be overrepresented in the qualitative subsample, which may bias study findings in the positive direction favoring acupuncture.

### Health equity implications

Diverse safety net participants with PDN found group acupuncture to be helpful in treating their pain. Furthermore, they noted acupuncture had a positive impact on their stress levels and overall QOL. Study participants were primarily low income and many were non-U.S.-born. Participants faced multiple poverty-related barriers, including a lack of services and social supports, and living in high-stress situations such as unstable or overcrowded housing. Attending acupuncture treatments required considerable efforts, such as taking public transportation, being on their feet for extended periods, and spending money on transportation. Despite the barriers present in their lives, participants demonstrated a high level of motivation to seek ways to alleviate their symptoms. Acupuncture can have a substantial impact on health-related QOL in vulnerable populations managing chronic conditions. More research is warranted to explore the ways in which acupuncture or other complementary therapies could bolster patient empowerment and hope to improve health outcomes in marginalized populations.

## Abbreviations Used

PDN: painful diabetic neuropathy
QOL: quality of life
SD: standard deviation
